# Peculiarities of DRX in a Highly-Alloyed Austenitic Stainless Steel

**DOI:** 10.3390/ma14144004

**Published:** 2021-07-17

**Authors:** Pavel Dolzhenko, Marina Tikhonova, Rustam Kaibyshev, Andrey Belyakov

**Affiliations:** Laboratory of Mechanical Properties of Nanostructured Materials and Superalloys, Belgorod State University, 308015 Belgorod, Russia; dolzhenko_p@bsu.edu.ru (P.D.); tikhonova@bsu.edu.ru (M.T.); rustam_kaibyshev@bsu.edu.ru (R.K.)

**Keywords:** austenitic stainless steel, hot deformation, dynamic recrystallization, grain size, dislocation density

## Abstract

The features of discontinuous dynamic recrystallization (DRX) in a highly-alloyed austenitic stainless steel were studied at temperatures of 800 °C to 1100 °C. Hot deformation accompanied by DRX was characterized by an activation energy of 415 kJ/mol. The frequency of the sequential DRX cycles depended on the deformation conditions; and the largest fraction of DRX grains with small grain orientation spread below 1° was observed at a temperature of around 1000 °C and a strain rate of about 10^−3^ s^−1^. The following power law relationships were obtained for DRX grain size (D_DRX_) and dislocation density (*ρ*) vs. temperature-compensated strain rate (Z) or peak flow stress (σ_P_): D_DRX_ ~ Z^−0.25^, *ρ* ~ Z^0.1^, σ_P_ ~ D_DRX_^−0.9^, σ_P_ ~ *ρ*^1.4^. The latter, i.e., σ_P_ ~ *ρ*^1.4^, was valid in the flow stress range below 300 MPa and changed to σ_P_ ~ *ρ*^0.5^ on increasing the stress. The obtained dependencies suggest a unique power law function between the dislocation density and the DRX grain size with an exponent of −0.5.

## 1. Introduction

Hot working is a common treatment frequently applied to various structural steels and alloys. One of the most interesting and important phenomena accompanying thermo-mechanical treatment at elevated temperatures is dynamic recrystallization (DRX), which may result in desirable microstructure evolution providing the required combination of mechanical properties of the processed semi-products [1,2,3]. Depending on the type of metallic material, i.e., crystal lattice, impurity, alloying extent, stacking fault energy (SFE), phase content, etc., various mechanisms of DRX contribute to the final microstructure. Discontinuous DRX involving cyclic nucleation and growth of new DRX grains commonly develops during hot working of face-centered cubic metals and alloys with low-to-medium SFE. The mean grain size in discontinuously DRX microstructures depends on the deformation conditions, namely, temperature and strain rate, similar to the flow stress, and can be related to the flow stress or temperature-compensated strain rate through power law functions [3,4,5]. A decrease in temperature and/or increase in strain rate results in an increase in the flow stress while decreasing the DRX grain size.

Austenitic steels are typical representatives of metallic materials undergoing discontinuous DRX under hot working conditions [6,7]. The main regularities of discontinuous DRX in single phase austenitic steels are well established to enable microstructure control in the steels directly during hot working. Austenitic stainless steels can be given as an example of materials that are successfully processed by thermo-mechanical treatments involving discontinuous DRX [8]. Good corrosion resistance of chromium–nickel austenitic stainless steels results in their wide application. It should be noted that single phase austenitic stainless steels with recrystallized microstructures are characterized by relatively low yield strength [9]. On the other hand, certain engineering devices/constructions require corrosion resistant materials with enhanced strength properties along with sufficient plasticity. The general approach to strengthen the steels involves complicated alloying in order to realize a solid solution and dispersed strengthening. In contrast to single phase austenite, however, the DRX behavior in multiphase steels and alloys including those strengthened by dispersed precipitations has not been studied in sufficient detail. The presence of secondary phases should alter the DRX behavior and append specific peculiarities untypical for ordinary DRX in single phase austenite [10]. DRX in multiphase austenite should be studied in close relation to phase content, taking into account any possible changes in secondary phase distribution. The aim of the present study was to investigate the features of discontinuous DRX during hot deformation of a highly-alloyed austenitic stainless steel, the chemical content of which is designed for low temperature applications.

## 2. Materials and Methods

The measured chemical content of experimental austenitic stainless steel includes 0.03% C, 0.4% N, <0.014% Si, 6% Mn, <0.005% P, <0.003% S, 21% Cr, 1.85% Mo, 10% Ni, 0.4% Nb (all in wt%). The steel samples were homogenized at 1000 °C for 8 h followed by multiple hot rolling at 1100 °C with pass strain of 10% to total reduction of 60% (i.e., a von Mises equivalent strain close to 1) in order to obtain a uniform initial microstructure composed of equiaxed recrystallized grains. The cylindrical specimens of ∅10 mm and 20 mm in height were subjected to isothermal compression tests to a strain of 1 followed by water quenching. A water jet was passed over the specimen just after deformation ceased to fix the DRX microstructure and prevent the development of any post-dynamic recrystallization process as much as possible. The specimens were compressed at temperatures of 800 to 1100 °C at initial strain rates of 10^−4^ to 10^−2^ s^−1^ by using an Instron 300XL testing machine with boron nitride as a lubricant.

The phase content was studied by X-ray diffraction using a Rigaku Ultima IV diffractometer with Cu Kα radiation (dwell time and scanning rate were 1.2 s and 1°/min, respectively) with supplementation by calculations using ThermoCalc software with TCFE7 database. The microstructural observations were carried out at the center portion of the compressed specimens using a Jeol JEM-2100 transmission electron microscope (TEM) and a Quanta 600 FEG scanning electron microscope (SEM) equipped with an automatic electron backscattering diffraction (EBSD) analyzer, incorporating an orientation imaging microscopy (OIM) system with TSL OIM Analysis 6 software. The OIM images were subjected to post-processing clean up treatment setting a minimal number of measured points per grain of 3. The points indexed with confidence index of less than 0.1 were removed from the analysis. They are shown by black points in the figures presented in the paper. The grain size was evaluated as an average of long and short intercepts counting all high-angle boundaries with misorientations of θ *≥* 15° ignoring CSL ∑3^n^ twin boundaries. The dislocation densities were evaluated by means of kernel average misorientation (θ_KAM_), which was calculated with upper limit of 15°, as *ρ* = 2 θ_KAM_/(b d) [11], where b and d are the Burgers vector and OIM the step size (d = 150 nm), respectively.

## 3. Results

### 3.1. Initial Microstructure

**Figure 1:**
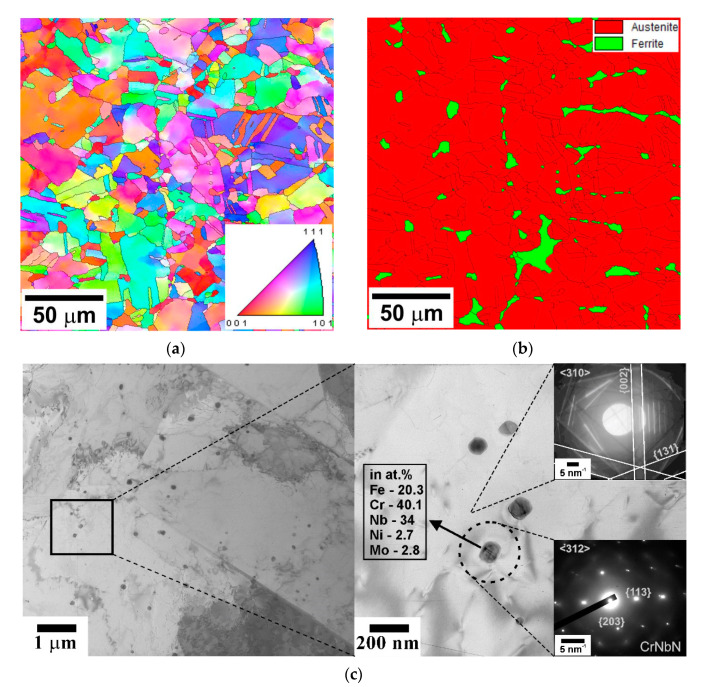
Initial microstructure of a highly-alloyed austenitic stainless steel; typical OIM micrograph (**a**), austenite/ferrite distribution (**b**), and Z-phase particle in the austenite matrix (**c**). High-angle grain boundaries are indicated by black lines in (**a**) and (**b**).

**Figure 2:**
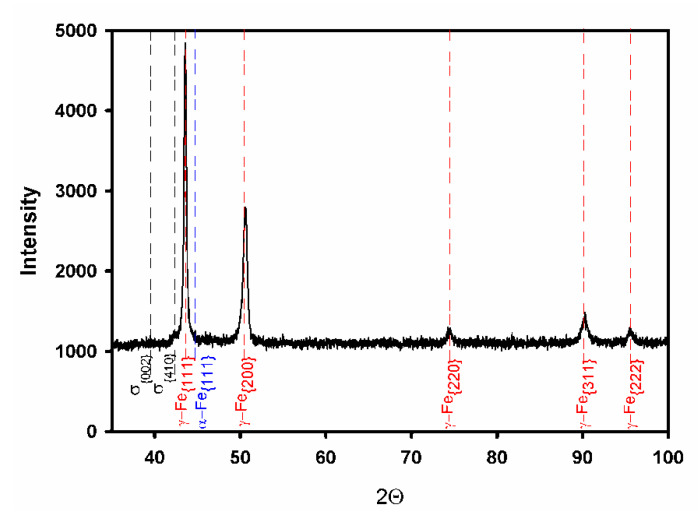
X-ray diffraction pattern of a highly-alloyed austenitic stainless steel annealed at 800 °C.

The initial microstructure is shown in Figure 1. The microstructure is mainly composed of austenite with a mean grain size of about 10 μm (Figure 1a). Besides austenite, the steel samples in the initial state include ferrite and dispersed particles of Z-phase (CrNbN). The former comprises about 10 vol% and appears as small portions between some austenite grains (Figure 1b). The size of Z-phase particles is about d = 100 nm (Figure 1c). According to ThermoCalc calculations, the fraction of these particles is about F_Z_ = 0.008 in the studied temperature range of 800–1100 °C. The corresponding nearest neighboring particle spacing in the volume can be evaluated as λ = 0.3 d (π/F_Z_)^1/3^ [12]; it comprises about 220 nm. In addition, ThermoCalc calculations predict the existence of a σ-phase (FeCr) at temperatures of T *≤* 900 °C and Cr_2_N at T *≤* 1000 °C. According to ThermoCalc calculations, the fraction of σ-phase increases from 0.033 to 0.14 with a decrease in temperature from 900 to 800 °C, whereas that of Cr_2_N increases from 0.006 at 1000 °C to 0.02 at 800 °C. The X-ray diffraction analysis of a specimen tested at 800 °C confirms the σ-phase formation (Figure 2), although its measured fraction does not exceed 0.002, whereas the fractions of Z-phase and Cr_2_N were not large enough to be revealed by X-ray diffraction.

### 3.2. Stress–Strain Behavior

**Figure 3:**
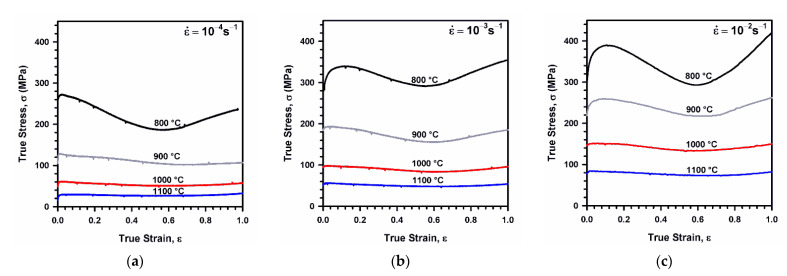
True stress vs. strain relationships for a highly-alloyed austenitic stainless steel subjected to isothermal compression at indicated temperatures and at strain rate of 10^−4^ s^−1^ (**a**), 10^−3^ s^−1^ (**b**), and 10^−2^ s^−1^ (**c**).

Typical true stress–strain curves obtained by isothermal compression tests are shown in Figure 3. The flow stresses demonstrate discontinuous DRX behavior. Namely, the flow stress increases to its maximum at an early deformation followed by strain softening upon further deformation. Note here, that such a decrease in the flow stress during compression may be associated with non-uniform deformation in addition to DRX development, especially, at 800 °C, when the strain localization leads to an ellipse-like cross section of compressed specimens with an aspect ratio above 1.3. In large strains above 0.6, the flow stress tends to increase. This stress increase is more pronounced at lower temperatures and can be attributed to increasing the friction contribution to the compression stress as the ratio of specimen height to diameter decreases. The level of flow stress increases while the deformation temperature decreases and/or strain rate increases. The strain corresponding to the peak flow stress, which can be used as a sign of DRX development, decreases as temperature increases and/or strain rate decreases, indicating DRX acceleration with temperature.

**Figure 4:**
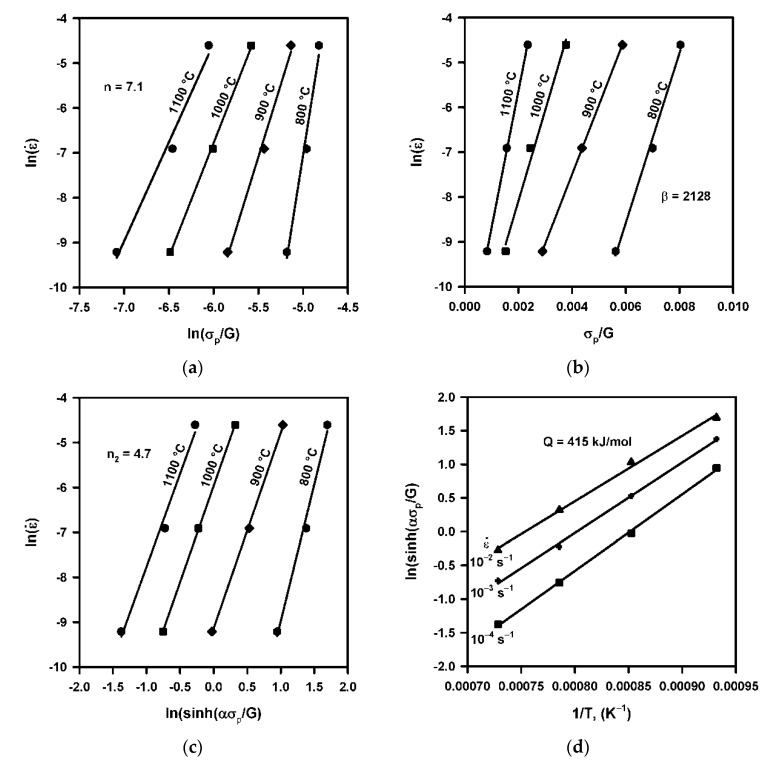
Relationships between strain rate and peak flow stress according to Equation (1) (**a**), Equation (2) (**b**), Equation (3) (**c**), and ln(sinh(ασ_P_/G)) vs. 1/T (**d**) for hot compression of a highly-alloyed austenitic stainless steel.

Formal analysis of deformation behavior can be carried out using the following relationships between the flow stress and strain rates [7].

(1) $$\dot{\varepsilon }=A{\left(\frac{\sigma }{G}\right)}^{n}exp\left(\frac{-Q}{RT}\right),$$

(2) $$\dot{\varepsilon }=A_{1}exp\left(\beta \frac{\sigma }{G}\right)exp\left(\frac{-Q}{RT}\right),$$

(3) $$\dot{\varepsilon }=A_{2}{\left(\sinh\left(\alpha \frac{\sigma }{G}\right)\right)}^{n_{2}}exp\left(\frac{-Q}{RT}\right),$$

where *A*, *A*_1_, *A*_2_, n, *β*, *α ≈ β*/n, n_2_ are material constants, *G* is the shear modulus, *Q* is the activation energy for deformation, *R* is the universal gas constant, and *T* is the temperature. Taking into account the temperature dependence of the shear modulus [13], the experimental relationship between the peak flow stress, strain rate, and temperature were plotted as shown in Figure 4 referring to Equations (1)–(3). Then, an activation energy of *Q* = 415 kJ/mol was obtained for hot deformation accompanied by discontinuous DRX under the studied conditions. The obtained activation energy is about 1.5 times more than that of volume self-diffusion [13]. Note here, that an increase in alloying extent increases the activation energy for hot deformation accompanied by DRX [14]. The relatively large *Q* value obtained in the present study can be attributed to the large amount of alloying elements in the studied steel. 

### 3.3. Deformation Microstructures

**Figure 5:**
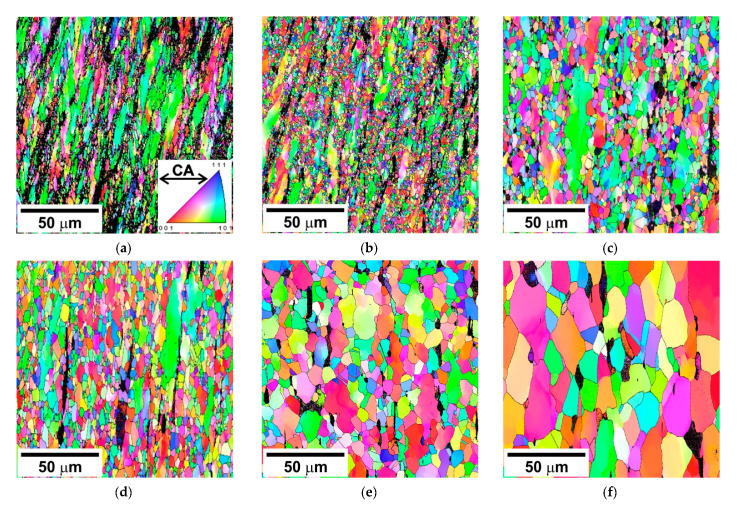
Typical OIM micrographs of a highly-alloyed austenitic stainless steel subjected to hot compression at 800 °C, 10^−2^ s^−1^ (**a**) 900 °C, 10^−2^ s^−1^ (**b**), 900 °C, 10^−4^ s^−1^ (**c**), 1000 °C, 10^−2^ s^−1^ (**d**), 1000 °C, 10^−4^ s^−1^ (**e**), and 1100 °C 10^−4^ s^−1^ (**f**). The colors indicate the direction along the compression axis (CA). The high-angle boundaries are indicated by black lines.

The specific deformation austenite microstructures which evolved in the present steel samples during the hot compression tests are shown in Figure 5. An increase in temperature and/or decrease in strain rate enlarged both the DRX fraction and the DRX grain size. A quite small DRX fraction was obtained after compression at 800 °C and at a strain rate of 10^−2^ s^−1^ (Figure 5a). In contrast, complete DRX can be observed in the specimens compressed at 1000 °C and at a strain rate below 10^−2^ s^−1^ (Figure 5e) or at 1100 °C irrespective of strain rate within the studied range (Figure 5f). Commonly, non-recrystallized portions are characterized by a fiber texture of <110> parallel to the compression axis (CA), the green color in Figure 5, whereas DRX grains exhibit various orientations with <100>//CA as the most frequent one, the red color in Figure 5, that is very similar to primary recrystallization [15,16].

**Figure 6:**
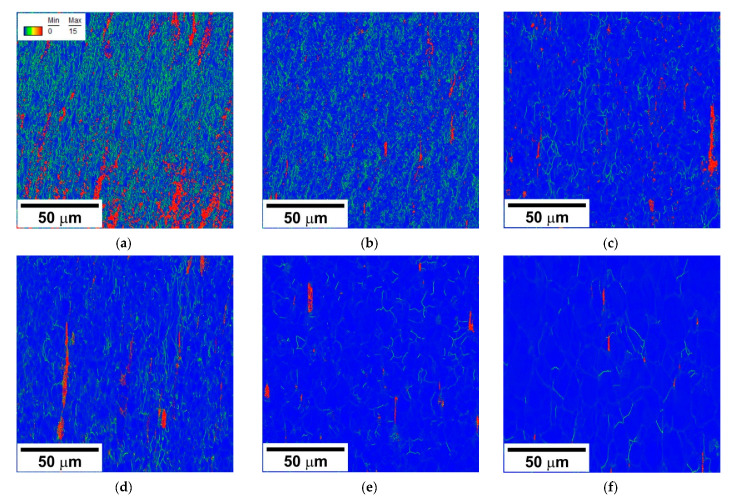
Kernel average misorientations in a highly-alloyed austenitic stainless steel subjected to hot compression at 800 °C, 10^−2^ s^−1^ (**a**) 900 °C, 10^−2^ s^−1^ (**b**), 900 °C, 10^−4^ s^−1^ (**c**), 1000 °C, 10^−2^ s^−1^ (**d**), 1000 °C, 10^−4^ s^−1^ (**e**), and 1100 °C 10^−4^ s^−1^ (**f**).

The deformation microstructures are characterized by a rather uniform distribution of kernel average misorientations (θ_KAM_) irrespective of the difference in DRX fraction in the various specimens (Figure 6). Large θ_KAM_ in Figure 6 are associated with dislocation sub-boundaries and, therefore, can be used as indicators of deformation substructure. The number density of such sub-boundaries decreases as strain rate decreases and/or deformation temperature increases which reflects the qualitatively same dependence of both DRX grain size and DRX subgrain size on the deformation conditions.

**Figure 7:**
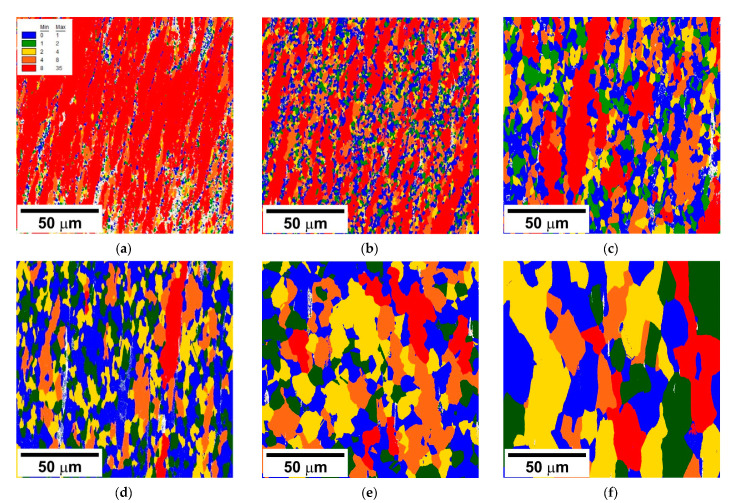
Grain orientation spread in a highly-alloyed austenitic stainless steel subjected to hot compression at 800 °C, 10^−2^ s^−1^ (**a**) 900 °C, 10^−2^ s^−1^ (**b**), 900 °C, 10^−4^ s^−1^ (**c**), 1000 °C, 10^−2^ s^−1^ (**d**), 1000 °C, 10^−4^ s^−1^ (**e**), and 1100 °C 10^−4^ s^−1^ (**f**).

The DRX development can be observed through the variation in the grain orientation spread (θ_GOS_ distribution in Figure 7). The work hardened grains with large θ_GOS_ above 8° dominate in the microstructure being evolved at relatively low temperatures below 900 °C (red color in Figure 7). The newly developed DRX nuclei and grains are characterized by small θ_GOS_ below 1° (blue color in Figure 7). It interesting to note that the fraction of grains with θ_GOS_ ≤ 1° does not exhibit direct dependence on temperature and/or strain rate. The largest fraction of such grains is observed after compression at 1000 °C and at a strain rate of 10^−2^ s^−1^ in Figure 7d, whereas a decrease in strain rate or an increase in temperature leads to an apparent decrease in the fraction of low θ_GOS_ grains (Figure 7e,f). The large fraction of low θ_GOS_ grains corresponds to the high frequency of discontinuous DRX cycles as suggested in previous studies [14]. On the other hand, the DRX microstructures may contain grains with rather large θ_GOS_ above 8° such as those in Figure 7e,f.

**Figure 8:**
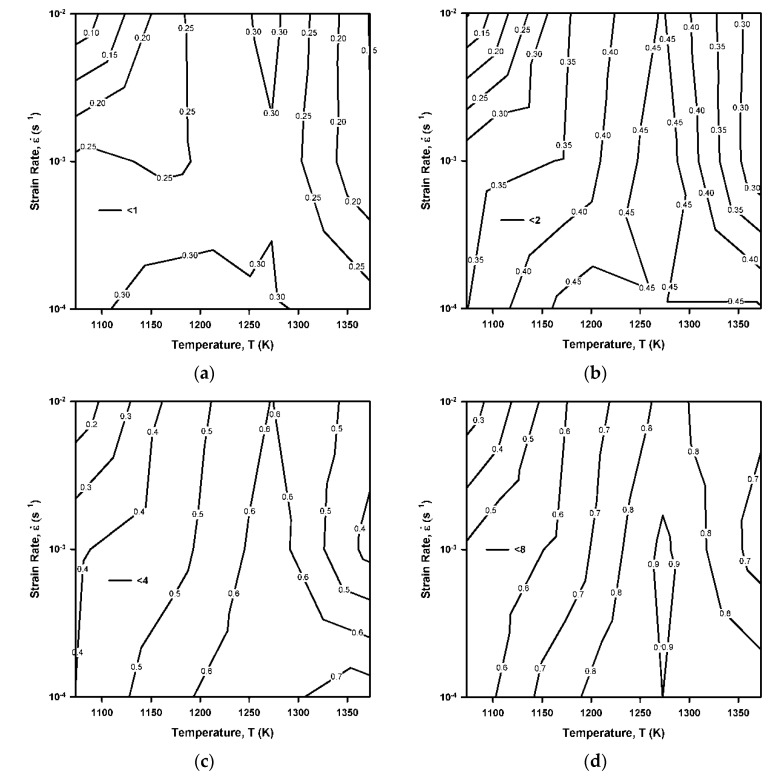
Fraction of grains with the grain orientation spread below 1 (**a**), 2 (**b**), 4 (**c**), and 8 (**d**) in a highly-alloyed austenitic stainless steel subjected to hot compression.

The effect of deformation conditions, i.e., temperature and strain rate, on the area fractions of grains with θ_GOS_ below 1°, 2°, 4°, or 8° is represented in Figure 8. Each map with contour lines of the same DRX fraction in Figure 8 envelops 12 different experimental temperature–strain rate data points. Generally, the area fraction of certain grains increases with an increase in critical angle of θ_GOS_. However, all the distributions in Figure 8 are characterized by the same manner of the temperature/strain rate dependence irrespective of critical θ_GOS_. Namely, the largest area fraction of grains with θ_GOS_ below some critical value is observed at certain deformation conditions, i.e., around 1000 °C and a strain rate of 10^−3^ s^−1^. Evidently, these deformation conditions correspond to the most frequent development of sequential cycles of discontinuous DRX in the present study.

**Figure 9:**
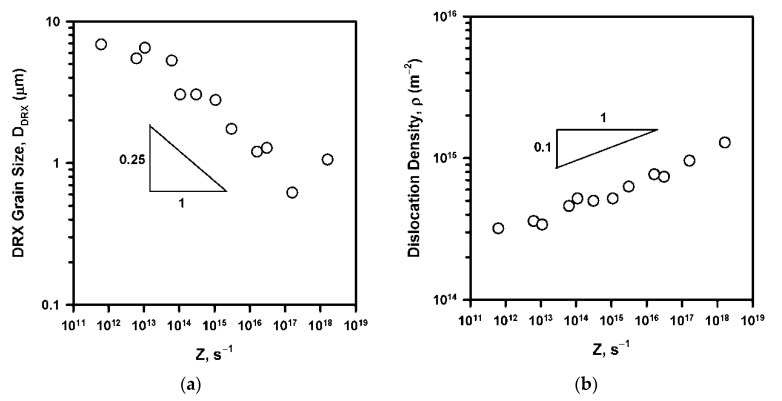
Effect of deformation conditions (Z) on the DRX grain size (**a**) and dislocation density (**b**).

The main parameters of DRX microstructures are commonly related to deformation conditions represented by the temperature-compensated strain rate, i.e., the Zener–Hollomon parameter, $Z=\dot{\varepsilon }\;expQ/RT$. Both the DRX grain size and the dislocation density obey power law functions of Z with exponents of −0.25 (Figure 9a) and 0.1 (Figure 9b), respectively. Note here, that in the case of uncompleted DRX (relatively low temperatures and high strain rates), the DRX grain size was measured in arbitrary selected areas composed of fine grains (several DRX areas were observed to collect a total of at least 300 DRX grains per each data point), while the dislocation density was calculated as a function of average θ_KAM_ in the observed OIM micrographs. Similar relationships have been observed in other studies on DRX in austenite [14,17,18,19,20]. The values of exponents of 0.2–0.4 and 0.12–0.2 were obtained for Z dependencies of DRX grain size [14,17,18,19,20] and dislocation density [19,20] evolved in austenitic steels under hot working conditions.

## 4. Discussion

**Figure 10:**
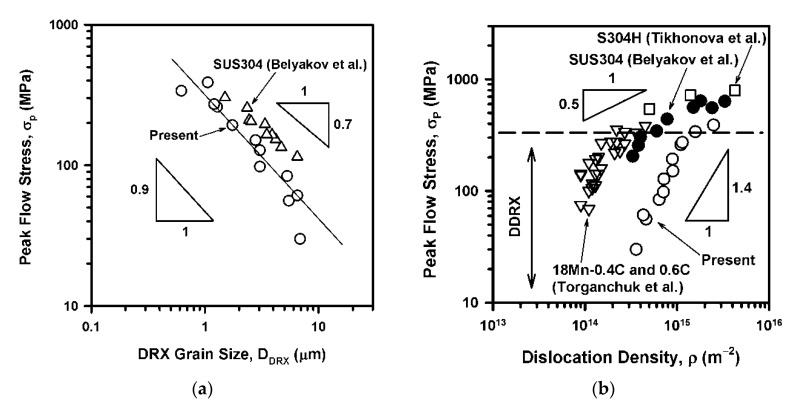
Relationships between the DRX grain size and the peak flow stress (**a**), and the dislocation density and the peak flow stress (**b**) in a highly-alloyed austenitic stainless steel subjected to hot compression. Some available data for austenitic steels [14,17,21] are also indicated for reference.

The power law relationships between the temperature-compensated strain rate (Z) and the flow stress as well as between Z and the DRX microstructures developed under hot working conditions allow us to relate the parameters of DRX microstructures to the flow stress. Commonly, the peak flow stress can be expressed by the DRX grain size with a grain size exponent of about −0.7. The relationship between the peak flow stress (σ_P_) and the DRX grain size (D_DRX_) for the present steel is shown in Figure 10a. The σ_P_–D_DRX_ relationship obtained for single-phase austenitic steel [21] is also indicated in the figure as a reference. Comparing to single phase steel, the present one exhibits finer DRX grains. Moreover, in contrast to other single phase austenitic steels, the present one is characterized by an exponent of about −0.9 in the corresponding power law function. The difference in the grain size can be attributed to the dispersed particles, which are closely spaced at 220 nm in the initial steel samples. The particles provide pinning pressure retarding the grain boundary motion. The pinning effect is more valuable for coarser microstructure, when the particle spacing becomes much smaller than the grain size and the DRX grain growth is accompanied by a decrease in its driving pressure.

The development of discontinuous DRX under hot working conditions is caused by work hardening very similar to primary recrystallization. Namely, the high dislocation density in work hardened grains provides driving pressure for the nucleation and growth of DRX grains. In contrast to static primary recrystallization, however, the DRX development does not remove the dislocations completely [22,23,24]. The cyclic character of discontinuous DRX results in a certain level of dislocation substructure that should depend on the deformation conditions or the flow stress. The dislocation substructures were considered as supplemental strengthening in previous studies on DRX in austenitic steels [19,25]. The relationship between the dislocation density and the peak flow stress in the present DRX steel samples is shown in Figure 10b along with some available literature data for high-Mn and 304-type steels [14,17,21]. Note here, the dislocation density was evaluated by means of θ_KAM_ in the high-Mn steel and by counting individual dislocations on TEM images for the 304-type steels. It is clearly seen in Figure 10b that the peak flow stress can be related to dislocation density through a power law function with an exponent of 1.4 in the range of flow stresses below about 300 MPa which corresponds to hot working accompanied by discontinuous DRX irrespective of the difference in the steel types and the calculation methods. Another interesting issue in Figure 10b is a tendency of the exponent to decrease to about 0.5 as stress increases. The deformation domain with flow stress above 300 MPa corresponds to warm-to-cold working, where the microstructure evolution is controlled by continuous DRX and other equations are valid for structure–property relationships [23]. The exponent value of 0.5 suggests a Taylor-type relationship between the stress and dislocation density in the case of warm/cold deformation, although this interesting phenomenon should be detailed in further investigations.

**Figure 11:**
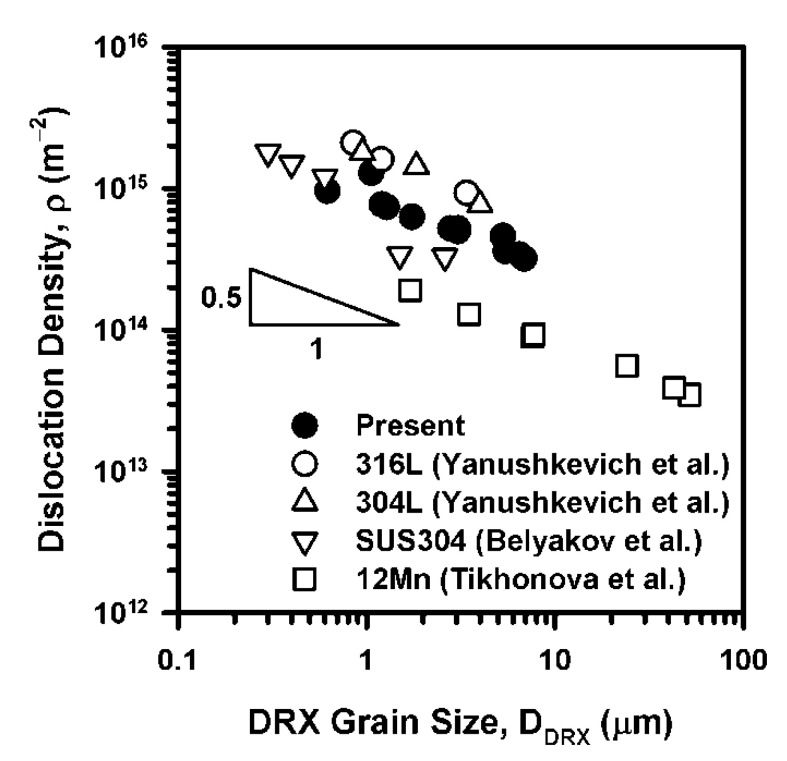
Relationship between the DRX grain size and the dislocation density in a highly-alloyed austenitic stainless steel subjected to hot compression along with some reference data [19,21,26].

The relationships in Figure 9 and Figure 10 suggest a power law function for the dislocation density versus the DRX grain size with an exponent of −0.5 for the present steel subjected to hot deformation accompanied by discontinuous DRX (Figure 11). It should be noted that similar relationships between the grain size and dislocation density were reported for other austenitic steels subjected to rolling under conditions of warm-to-hot working [19,21,26]. Although the grain size exponent of −0.6 was reported in previous papers, this value is quite close to −0.5 in Figure 11 and seems to reflect a unique relationship, which is valid for all DRX microstructures. Moreover, the revealed relationships between the flow stress, the grain size, and the dislocation density are applicable for deformation microstructures with different DRX fractions and different DRX kinetics.

## 5. Conclusions

The discontinuous DRX behavior was studied for a highly-alloyed austenitic stainless steel at temperatures of 800 °C to 1100 °C. The main results can be summarized as follows:

1. The deformation behavior was characterized by an activation energy of 415 kJ/mol and was accompanied by discontinuous DRX with the most frequent cycles during deformation at temperature and strain rate of approx. 1000 °C and 10^−3^ s^−1^, respectively.
2. Both the DRX grain size and the dislocation density could be expressed by power law functions of temperature-compensated strain rate, Z, with exponents of −0.25 and 0.1, respectively.
3. Analogously, a power law function was obtained for the peak flow stress and the DRX grain size with a grain size exponent of −0.9. The peak flow stress in the range below about 300 MPa, i.e., under hot working conditions, could be related to the dislocation density with a power law function with an exponent of 1.4, which tended to decrease to about 0.5 on increasing the flow stress to well above 300 MPa, i.e., in the range of cold-to-warm working.
4. The obtained stress dependencies for the DRX grain size and dislocation densities result in a unique power law function for the dislocation density versus the DRX grain size with an exponent of −0.5.
5. The revealed relationships between the flow stress, the grain size, and the dislocation density are applicable for deformation microstructures with different DRX fractions that evolved over a wide range of deformation conditions with various DRX kinetics.

## Data Availability

The data presented in this study are available on request from the corresponding author.

## References

[1] Jonas J.J., Sellars C.M., Tegart W.J. (1969). Strength and structure under hot-working conditions. Metall. Rev..

[2] McQueen H.J., Jonas J.J., Arsenault R.J. (1975). Recovery and Recrystallization during High Temperature Deformation. Treatise on Materials Science and Technology.

[3] Sakai T., Jonas J.J. (1984). Dynamic recrystallization: Mechanical and microstructural considerations. Acta Metall..

[4] Derby B. (1991). The dependence of grain size on stress during dynamic recrystallization. Acta Metall. Mater..

[5] Sakai T. (1995). Dynamic recrystallization microstructures under hot working conditions. J. Mater. Process. Technol..

[6] Maki T., Akasaka K., Okuno K., Tamura I. (1982). Dynamic recrystallization of austenite in 18-8 stainless steel and 18 Ni maraging steel. Trans. Iron Steel Inst. Jpn..

[7] McQueen H.J., Ryan N.D. (2002). Constitutive analysis in hot working. Mater. Sci. Eng. A.

[8] Tikhonova M., Kaibyshev R., Belyakov A. (2018). Microstructure and mechanical properties of austenitic stainless steels after dynamic and post-dynamic recrystallization treatment. Adv. Eng. Mater..

[9] Lo K.H., Shek C.H., Lai J.K.L. (2009). Recent developments in stainless steels. Mater. Sci. Eng. R.

[10] Huang K., Logé R.E. (2016). A review of dynamic recrystallization phenomena in metallic materials. Mater. Des..

[11] Calcagnotto M., Ponge D., Demir E., Raabe D. (2010). Orientation gradients and geometrically necessary dislocations in ultrafine grained dual-phase steels studied by 2D and 3D EBSD. Mater. Sci. Eng. A.

[12] Humphreys F.J., Hatherly M. (2004). Recrystallization and Related Annealing Phenomena.

[13] Frost H.J., Ashby M.F. (1982). Deformation Mechanism Maps.

[14] Torganchuk V., Rybalchenko O., Dobatkin S.V., Belyakov A., Kaibyshev R. (2020). Hot Deformation and Dynamic Recrystallization of 18%Mn Twinning-Induced Plasticity Steels. Adv. Eng. Mater..

[15] Hutchinson B., Nes E. (1992). Texture development during grain growth—A useful rule-of-thumb. Mater. Sci. Forum.

[16] Engler O., Vatne H.E., Nes E. (1996). The roles of oriented nucleation and oriented growth on recrystallization textures in commercial purity aluminium. Mater. Sci. Eng. A.

[17] Tikhonova M., Belyakov A., Kaibyshev R. (2013). Strain-induced grain evolution in an austenitic stainless steel under warm multiple forging. Mater. Sci. Eng. A.

[18] Yanushkevich Z., Lugovskaya A., Belyakov A., Kaibyshev R. (2016). Deformation microstructures and tensile properties of an austenitic stainless steel subjected to multiple warm rolling. Mater. Sci. Eng. A.

[19] Tikhonova M., Torganchuk V., Brasche F., Molodov D.A., Belyakov A., Kaibyshev R. (2019). Effect of warm to hot rolling on microstructure, texture and mechanical properties of an advanced medium-Mn steel. Metall. Mater. Trans. A.

[20] Torganchuk V., Morozova A., Tikhonova M., Kaibyshev R., Belyakov A. (2019). Grain sizes and dislocation densities in fcc-metallic materials processed by warm to hot working. J. Phys. Conf. Ser..

[21] Belyakov A., Sakai T., Miura H., Kaibyshev R. (1999). Grain refinement under multiple warm deformation in 304 type austenitic stainless steel. ISIJ Int..

[22] Sakai T., Ohashi M. (1990). Dislocation substructures developed during dynamic recrystallization in polycrystalline nickel. Mater. Sci. Technol..

[23] Sakai T., Belyakov A., Kaibyshev R., Miura H., Jonas J.J. (2014). Dynamic and post-dynamic recrystallization under hot, cold and severe plastic deformation conditions. Prog. Mater. Sci..

[24] Chen D.-D., Lin Y.C., Wu F. (2019). A design framework for optimizing forming processing parameters based on matrix cellular automaton and neural network-based model predictive control methods. Appl. Math. Modell..

[25] Dolzhenko P., Tikhonova M., Kaibyshev R., Belyakov A. (2019). Dynamically recrystallized microstructures, textures, and tensile properties of a hot worked high-Mn steel. Metals.

[26] Yanushkevich Z., Dobatkin S.V., Belyakov A., Kaibyshev R. (2017). Hall–Petch relationship for austenitic stainless steels processed by large strain warm rolling. Acta Mater..

